# Editorial: Cardiac Fibrosis, From Lineage Tracing to Therapeutic Application

**DOI:** 10.3389/fphys.2020.641771

**Published:** 2021-01-20

**Authors:** Claudio de Lucia, Markus Wallner, Domenico Corradi, Gianluigi Pironti

**Affiliations:** ^1^Center for Translational Medicine, Temple University, Philadelphia, PA, United States; ^2^Division of Cardiology, Medical University of Graz, Graz, Austria; ^3^Cardiovascular Research Center, Temple University, Philadelphia, PA, United States; ^4^Center for Biomarker Research in Medicine, CBmed GmbH, Graz, Austria; ^5^Unit of Pathology, Department of Medicine and Surgery, University of Parma, Parma, Italy; ^6^Cardiology Research Unit, Department of Medicine, Karolinska Institute Stockholm, Solna, Sweden

**Keywords:** cardiac fibrosis, myofibroblast, cardiac fibroblast, reparative fibrosis, reactive fibrosis, anti-fibrotic therapy, lineage tracing

## What is Cardiac Fibrosis?

**Figure 1:**
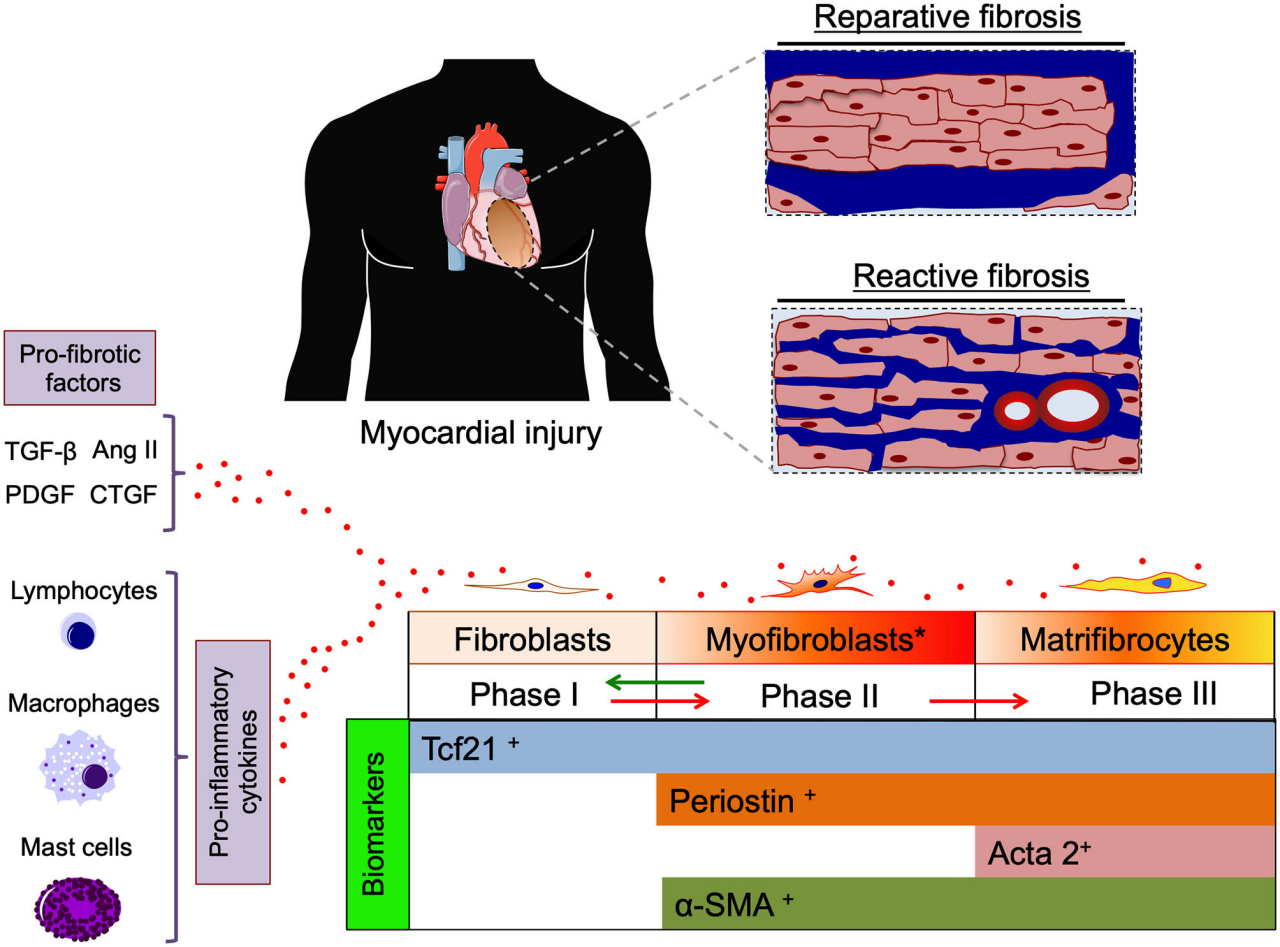
Cartoon depicting two pathogenic mechanisms of cardiac fibrosis, “reparative” (replacing dead cardiomyocytes) and “reactive” (interstitial and perivascular) cardiac fibrosis (blue color). Pro-fibrotic factors (TGF-β, AngII, PDGF, and CTGF) and cytokines released from inflammatory cells promote differentiation of fibroblasts toward activated myofibroblasts and matrifibrocytes actively producing extracellular matrix (ECM). Specific biomarkers of different stages of cardiac fibroblast differentiation are indicated on the bottom. TGF-β, transforming grow factor β; Ang II, angiotensin type II; PDGF, platelet derived grow factor; CTGF, connective tissue grow factor; Tcf21, transcription factor 21(biomarker for cardiac fibroblasts); Periostin, (biomarker of activated myofibroblasts) Acta2, smooth muscle actin alpha 2 (biomarker of matrifibrocytes); α-SMA, α smooth muscle actin.

Roughly 6% of healthy myocardium is composed of pure extracellular matrix (ECM) such as collagen fibers and, to a lesser extent, elastic fibers. This interstitial extracellular mixture—mainly synthesized by fibroblast and myofibroblasts—creates a three-dimensional cardiac skeleton which allows the cardiomyocytes to perform their contractile functions (Ten Tusscher and Panfilov, 2007). An excessive deposition of collagen fibers in the myocardium is commonly referred to as “fibrosis,” which is regulated by ECM production, activity of matrix metalloproteinases (MMPs) and their endogenous inhibitors (TIMPs) (De Boer et al., 2019; Frangogiannis, 2019). Various subsets of leukocytes play an important modulatory role determining the characteristics of the fibrotic response and cardiac remodeling post-injury (Frangogiannis, 2019). Disproportionate amounts of ECM (either focal or diffuse, scar-like, thin around single or small groups of muscle cells) represents an interstitial encumbrance which may lower myocardial compliance, decrease ventricular filling, interfere with electrical coupling, predispose to rhythm disturbances and ultimately lead to depressed cardiac function (Sharma and Kass, 2014; Nattel, 2017). Schematically, fibrosis may be secondary to two different—but not mutually exclusive—pathogenic mechanisms: (1). “reparative” fibrosis which replaces myocardial areas where cardiomyocytes have undergone cell death (i.e., ischemic events); and/or (2). “reactive” fibrosis which is driven by a series of stimuli (e.g., pressure overload, inflammation, metabolic dysfunction, aging) and mediators (e.g., AngII, PDGF, TGF-β, and CTGF) (Hanna et al., 2004; Corradi et al., 2008; De Boer et al., 2019; Frangogiannis, 2019) (Figure 1).

## Which Cell is To Blame?

The process of cardiac fibrosis can be schematically divided into three phases based on the cell type mainly involved: (i) resident quiescent cardiac fibroblasts inhabit myocardial tissue; (ii) during myocardial injury (MI), fibroblasts differentiate into myofibroblasts, which actively proliferate and secrete ECM until the injury is resolved; (iii) finally the site of injury is populated by not proliferative and terminally differentiated matrifibrocytes secreting ECM in order to maintain the integrity of the scar (Eschenhagen, 2018; Fu et al., 2018) (Figure 1).

Fu et al. described state-of-the-art techniques to trace the lineage of fibrogenic cells following cardiac injury, providing tools to specifically trace the different stages of cardiac fibrosis. Although the high grade of plasticity makes the lineage tracing of cardiac fibroblasts quite challenging, some biomarkers are known to uniquely identify fibroblasts (i.e., Tcf21), activated myofibroblasts (i.e., Periostin), and matrifibrocytes (i.e., ACTA2) (Fu et al., 2018). In terms of cellular signaling the activation of TGF-β receptor Smad2-3 pathway seems to be the principal mediator of myofibroblast activation and ECM accumulation.

Shi et al. demonstrated that Notch3 is involved in the regulation of cardiac fibrosis influencing fibroblast proliferation and myofibroblast transition/apoptosis via RhoA/ROCK/Hif1-α signaling pathway inhibition.

Ni et al. discovered that diabetic cardiomyopathy might benefit from icariin (a flavonoid monomer isolated from the herb Epimedium) treatment, which reduced cardiac fibrosis and ameliorated cardiomyocytes mitochondrial function through Apelin/Sirt3 pathway. Zhao et al. described how muscarinic acetylcholine receptor 3 (M3R) signaling after choline activation represents another important axes that controls cardiac fibrosis through TGF-β1/Smad2-3/p38 MAPK pathway.

Thomas and Grisanti reviewed the extensive crosstalk between inflammation and cardiac fibrosis contributing to the progression of heart failure (HF). Following myocardial injury and cardiomyocytes death, local inflammatory cells (i.e., mast cells, B and T cells and macrophages) infiltrate the site of injury and secrete pro-inflammatory mediators (i.e., TNF-α, IL-1-β, IL-6), which play an important regulatory role in the transition from quiescent resident fibroblasts into active and proliferative myofibroblasts, initiating the production of ECM components. Narrowing the focus to one specific mediator in the crosstalk between the inflammatory response and development of fibrosis, Okyere and Tilley described the role of leukocytes in the regulation of cardiac remodeling. Both innate and adaptive leukocytes critically influence pathological fibrotic remodeling.

Interestingly, cardiac fibrosis is regulated over an organized intercellular communication, where extracellular vehicles (EVs) play a crucial role. The interplay among macrophages, fibroblasts, and endothelial cells represents a major driving force of myocardial fibrosis. Rogers et al. described a cell-free therapeutic application based on EVs secreted from stem/progenitor cells that can directly stimulate the trans-differentiation of pro-inflammatory M1 macrophages to anti-inflammatory M2 macrophages(Silva et al., 2017), thereby reducing fibrosis in preclinical models of heart failure.

Marek-Iannucci et al. established a minimally invasive and cost-effective model of cooling pericardial perfusion in swine, which highlights the potential therapeutic effects of hypothermia post-ischemia/reperfusion injury in reducing cardiac fibrosis, inflammation and immune cell recruitment.

Farini et al. showed that cardiac expression of the inflammatory mediator Pentraxin 3 (PTX3) influences inflammatory/fibrotic pathways in an animal model of Duchenne Muscular Dystrophy and may be an interesting therapeutic target.

## When is The Right Time To Block Fibrosis?

Degradation of large areas of replacement fibrosis could be catastrophic unless accompanied by robust cardiac regeneration, due to the negligible endogenous regenerative potential of the adult heart. Upon cardiac injury, the compensatory but maladaptive fibrotic response mediated by cardiac fibroblast can vary significantly among different injury types (Khalil et al., 2019). While the beneficial effect of fibrotic tissue may outweigh its deleterious effect after an acute injury that causes massive cardiomyocyte death (i.e., reparative fibrosis in a myocardial infarction), the effects of reactive interstitial fibrosis in chronic conditions may be largely detrimental. The timing is of utmost importance since a too early intervention can cause adverse effects on wound healing, enhancing LV rupture and increasing the mortality rate in HF patients. Based on this, any medical and surgical treatment should be aimed at blocking/mitigating the excessive ECM deposition between single cardiomyocytes and/or the fine interstitial fibrosis around scar-like sclerotic areas replacing significant cardiomyocytes necrotic cell losses.

Ma et al. described a preclinical model of left atria (LA) fibrosis with increased arrhythmogenesis following isoproterenol (ISO) injections for 5 weeks in rats. The anti-fibrotic and anti-inflammatory agent pirfenidone administered 1 week after completion of ISO decreased LA fibrosis and arrhythmia leading to an improvement in cardiac function.

De Simone et al. showed that cardiac myofibroblast are not excitable cells, electronically coupled to cardiomyocytes and their function is not limited solely to ECM production and protecting the architecture of the heart, but is also important for regulating the cardiac electrical conduction. After excessive collagen deposition, only scarce cellular structures populate the sclerotic area, which likely is not capable of creating electrically efficient fibroblast/cardiomyocyte couplings and causing arrhythmogenesis(Callegari et al., 2020).

Shang et al. presented preclinical and clinical evidences linking together elevated levels of β1AR autoantibodies, circulating fibrosis markers and atrial remodeling in patients with paroxysmal atrial fibrillation (AF). Overexpressing β1AR antibodies, which act as βAR agonists, impacted electrophysiological properties in terms of atrial effective refractory period (AERP), AF inducibility, and electrical conduction in rabbits.

## Conclusions

The implication of early delivery of therapies post-MI will most certainly differ from the treatment for non-ischemic diastolic dysfunction with a stiff left ventricle and even more so from the treatment of end-stage heart failure with more pronounced fibrosis. While the fibrotic response following myocardial injury may initially be compensatory, could eventually be maladaptive if the large cardiac functional reserve is compromised. Therapeutic intervention in this setting should aim to prevent the fine sclerotic tissue deposition between the cardiomyocytes. This could be implemented with personalized anti-fibrotic therapy with specific timing of the intervention and tailoring it to the type of injury rather than completely blocking the fibrosis pathway.

## Author Contributions

GP substantially contributed to the conception and design of the editorial, literature search, drafting the article, and revising the article critically for important intellectual content. CL contributed in literature search, drafting the article, and revising the article critically for important intellectual content. MW contributed in literature search, drafting the article, and revising the article critically for important intellectual content. DC contributed in literature search and drafting the article and revising the article critically for important intellectual content. All the authors approved the final version of the article to be published.

## Conflict of Interest

MW was employed by the company Center for Biomarker Research in Medicine, CBmed GmbH, Austria. The remaining authors declare that the research was conducted in the absence of any commercial or financial relationships that could be construed as a potential conflict of interest.

## Abbreviations

TIMPs: tissue inhibitors of metalloproteases
AngII: Angiotensin type II
PDGF: Platelet derived grow factor
TGF-β: Transforming grow factor β
CTGF: connective tissue grow factor
TNF-α: Tumor necrosis factor α
IL-1-β: Interleukin 1 β
IL-6: Interleukin 6
Tcf21: transcription factor 21
Acta2: smooth muscle actin alpha 2.
